# Cambial response of Norway spruce to modified carbon availability by phloem girdling

**DOI:** 10.1093/treephys/tpx077

**Published:** 2017-11-01

**Authors:** Andrea Winkler, Walter Oberhuber

**Affiliations:** Institute of Botany, Leopold-Franzens-University of Innsbruck, Sternwartestrasse 15, A-6020 Innsbruck, Austria

**Keywords:** cambial activity, carbon allocation, drought, Norway spruce, wood anatomy, xylem

## Abstract

We tested the hypothesis that increase in carbon (C) availability in Norway spruce saplings (*Picea abies* (L.) Karst.) intensifies cambial cell division and increases cell lumen diameter (CLD) and cell wall thickness (CWT) when water availability is adequate. To accomplish this, we experimentally subjected 6-year-old *P. abies* saplings (*n* = 80 trees) to two levels of soil humidity (watered versus drought conditions) and manipulated tree C status by physically blocking phloem transport at three girdling dates (GDs). Stem girdling occurred in mid-March (day of the year (doy) 77) and in mid-May (GD doy 138), i.e., ~4 weeks before the onset of bud break and during vigorous stem growth, respectively, and in early July (GD doy 190), i.e., 6 and 4 weeks after cessation of radial growth in drought-stressed trees and shoot growth in both soil humidity (SH) treatments, respectively. In response to phloem blockage a striking increase in the number of xylem cells at all GDs and reactivation of cambial activity in drought-stressed trees was detected above the girdling zone, while below girdling xylem formation stopped in both SH-treatments. Although girdling differently affected wood anatomical parameters (CLD, CWT and CLD:CWT ratio) during earlywood and latewood formation, GD had a minor effect on cambial cell division and xylem cell differentiation. Results also revealed that phloem girdling outweighed drought effects imposed on cambial activity. We explain our findings by accumulation of carbohydrates, osmotically active sugars and/or C based signaling compound(s) in response to girdling. Altogether, we conclude that wood formation in *P. abies* saplings is limited by C availability, which is most likely caused by high C demand belowground especially under drought.

## Introduction

Drought is the primary environmental factor that impairs forest primary production (Ciais et al. 2005) and reduces stem growth (e.g., Kramer 1983, Fritts 2001, Dobbertin 2005, Zweifel et al. 2006, Cailleret et al. 2017). Based on several tree ring studies conducted within dry inner Alpine environments, drought stress in spring limits radial growth of coniferous species (Oberhuber et al. 1998, Rigling et al. 2002, Eilmann et al. 2006, Schuster and Oberhuber 2013) and intra-annual dynamics of wood formation revealed that maximum growth rate peaks early during the growing season prior to occurrence of more favorable environmental conditions, i.e., repeated high rainfall events during summer (Gruber et al. 2010, Oberhuber et al. 2014). Plants are able to adjust carbon (C) allocation to optimize nutrient uptake under stress conditions, e.g., more C is allocated to roots when drought prevails (Cannell and Dewar 1994, Ericsson et al. 1996, Brunner et al. 2015, Hagedorn et al. 2016). Abramoff and Finzi (2015) reported that root and shoot growth are not synchronous and in temperate biomes shoot growth peaked 28 ± 12 days earlier than root growth. Hence, in drought-prone environments high C demand belowground might explain early culmination of aboveground stem growth.

Norway spruce (*Picea abies* (L.) Karst.) is the dominant coniferous tree species in the Central European Alps, and natural populations are found from the lower montane region up to the tree line (Ellenberg and Leuschner 2010). *Picea abies* dominates in areas where relatively low temperatures and high precipitation prevail, but is not well adapted to drought because its fine roots are distributed primarily in upper soil layers (Schmid and Kazda 2002, Puhe 2003). Hence, drought periods during the growing season lead to reduced growth and a shortened growing season (Mäkinen et al. 2001, Alavi 2002, Pichler and Oberhuber 2007, Lévesque et al. 2013, Swidrak et al. 2014) and young trees—including *P. abies*—were found to be especially vulnerable to limited water supply (Rossi et al. 2009, de Luis et al. 2011, Oberhuber et al. 2015a, Balducci et al. 2016).

Xylem tissue in coniferous wood is developed through a highly dynamic process encompassing cambial cell division, cell radial enlargement, secondary cell wall formation and lignification (the latter two processes are termed cell wall thickening hereafter) and programmed cell death (see reviews in Plomion et al. 2001, Scarpella and Meijer 2004). Wood formation comprises expansive and structural growth, i.e., irreversible increase in volume and accumulation of biomass into structure, respectively (Pantin et al. 2012). Accordingly, drought stress can affect wood formation directly through impairment of cell enlargement and cell wall thickening and during severe drought through inhibition of cambial cell division (e.g., Denne and Dodd 1981, Abe et al. 2003, Muller et al. 2011, Pantin et al. 2013).

The maintenance of an efficient water transport system is of particular importance under drought to avoid xylem cavitation or downregulation of photosynthesis (Bréda et al. 2006). Trees are able to adapt their water-conducting tissue to prevailing environmental conditions and it is well known that climate before or during the wood formation process affects wood properties, e.g., cell number, cell lumen diameter (CLD) and cell wall thickness (CWT) (for a review see Fonti et al. 2010). Several studies have highlighted a close relationship between wood-anatomical variables and seasonal climatic conditions in conifers (Yasue et al. 2000, Panyushkina et al. 2003, DeSoto et al. 2011, Liang et al. 2013). However, there is lack of knowledge regarding influence of C availability on wood formation, although cambial activity and wood cell development are strongly dependent on the availability of C compounds in the phases of cell enlargement and cell wall thickening (Oribe et al. 2003, Deslauriers et al. 2009, 2016, Simard et al. 2013) and ‘source’-limitation of wood formation may develop under prolonged drought stress (Rennenberg et al. 2006, Zweifel et al. 2007, Balducci et al. 2015).

The use of manipulative experiments is crucial to understand the influence of C availability on wood formation under different environmental conditions. Physical blockage of phloem transport through girdling is frequently applied to study effects of C status on tree growth (Wilson and Gartner 2002, Maier et al. 2010, Maunoury-Danger et al. 2010, López et al. 2015). Because wood formation depends on a continuous supply of carbohydrates, manipulation of the C status of the stem through physical manipulation of C availability by disruption of phloem transport can reveal C limitation of aboveground growth. Phloem blockage causes accumulation and depletion of carbohydrates above and below the girdle, respectively (Daudet et al. 2005, De Schepper et al. 2010, Maier et al. 2010) and girdling experiments have demonstrated the importance of current photoassimilate flux to sustain stem growth (Daudet et al. 2005).

The main focus of this study was to study effects of interrupted C flow at distinct phenological stages on cambial cell division and xylem cell differentiation in *P. abies* saplings under different soil water availability, i.e., watered vs drought-stressed. We hypothesized that C accumulation above the girdling zone intensified cambial cell division, and increased CLD and CWT, when water availability was adequate. Furthermore, no growth response was expected below the girdling zone and also above the girdling zone if girdling occurred after regular cambial activity has ceased. The results of this study contribute to the understanding of the early culmination of radial stem growth found in coniferous species at drought-prone sites (Gruber et al. 2010, Oberhuber et al. 2014) and the interaction between endogenous and environmental factors on wood formation.

## Materials and methods

### Plant material and treatments

The experiment was conducted at latitude 47°16′05″N, longitude 11°22′45″E, the Botanical Garden of the Institute of Botany, University of Innsbruck, Austria. In autumn 2013 6-year-old *P. abies* trees, previously grown outdoor in a tree nursery, with a stem height of ~135 cm and a diameter of ~3.5 cm at 5-cm stem height were replanted in 80-l containers (filled with fertilized garden soil above a drainage layer at the bottom of the container) and placed in a regular polytunnel with clear polythene cover (200 micron UV-stabilized film) to ensure similar microclimatic conditions for all trees under study. The trees were allowed to recover from transplant shock and adapt to conditions in the containers for one growing season. Starting in mid-March 2015, the trees were subjected to different soil moisture conditions, i.e., watered versus drought-stressed, and were divided into four subsets: the control (no phloem blockage) and a phloem blockage treatment at three girdling dates (GDs) (*n* = 10 trees in each subset). Watered trees were irrigated weekly to field capacity in early morning. Drought treatment was accomplished by watering less frequently (10–14 day intervals) and irrigated amount was half compared with watered trees. The experiment included a total of 80 trees (2 environmental settings × 4 subsets × 10 trees = 80 trees).

Air temperature and relative humidity (CS215 temperature and relative humidity sensor, Campbell Scientific, Shepshed, UK) and solar radiation (PhAR; LI-200S Pyranometer Sensor, Campbell Scientific) were continuously measured within the polytunnel at a height of 2m. The soil temperature at 10cm soil depth (T 107 Temperature Probe, Campbell Scientific) and the volumetric soil water content (SWC) in the uppermost 30 cm was recorded (CS616 Water Content Reflectometer, Campbell Scientific) for both the watered and drought-stressed trees (*n* = 10 per soil humidity (SH) treatment). All environmental data were recorded using a CR1000 data logger and three AM 16/32 multiplexers (Campbell Scientific) programmed to record 30-min averages of measurements collected every minute.

### Manipulation of C availability by means of double girdling

We applied double girdling to produce three distinct horizontal zones (upper (UZ), middle (MZ) and lower zone (LZ)) with respect to the phloem sap sucrose supply of the stem (see Figure S1 available as Supplementary Data at *Tree Physiology* Online; cf. De Schepper et al. 2010). While UZ and LZ could still receive carbohydrates from the leaves and roots, respectively, MZ was completely isolated and could only use existing local carbohydrate reserves to maintain metabolism and growth. Two 1- to 2-cm wide bands of bark (extending to the xylem) were carefully detached from the stem at a height of approximately 5 and 15 cm above the soil surface and the xylem tissue was covered with aluminum foil to prevent dehydration. Because concentration of sugars in phloem vary throughout the year and girdling also inhibits transport of shoot-derived growth substances necessary for cambium activity (Larson 1994), the trees were girdled at three phenological stages: (i) in mid-March 2015 (GD day of the year (doy) 77), i.e., several weeks before bud swelling and the onset of cambial activity; (ii) during vigorous earlywood and shoot growth in mid-May (GD doy 138); and (iii) after cessation of shoot growth in July (GD doy 190). Phenological stages were based on intra-annual dynamics of shoot and radial stem growth determined in mature *P. abies* trees (Swidrak et al. 2013). At GD doy 190 radial and shoot growth in drought-stressed trees has already stopped for approximately 6 and 4 weeks, respectively (cf. Figure 2).

The ‘pinning method’, i.e., the marking of the cambium by micro-injury using a small needle (diameter ~1 mm), was applied to all three zones to mark GDs during the study period (Gričar et al. 2007, Seo et al. 2007). Since the wound tissue separates cells formed before and after pinning, the wound reaction was used for exact dating of wood formation before and after phloem blockage.

### Determination of radial stem growth and shoot growth and wood anatomical parameters

Intra-annual dynamics of radial growth in watered and drought-stressed control trees was monitored by installing automated diameter dendrometers (*n* = 6 per SH-treatment; type DD-S, Ecomatik, Munich, Germany). Dead outermost layers (periderm) of the bark were slightly removed to ensure close contact with the stem. Growth of the terminal shoot of all trees (*n* = 10 trees per subset) was measured using a caliper.

At the end of the study period stem cross-sections were collected from all trees from the following locations: girdling zones UZ, MZ and LZ ~1 cm from the girdling zone to avoid regions with wound responses, and 50 cm above UZ. Total cell number, CWT and CLD were measured on stem transverse sections of ~20-μm thickness (*n* = 3–5 trees), which were cut using a rotary microtome and stained with a water solution of 0.05% cresyl fast violet. Cell parameters were determined by applying the image analysis software ProgRes Capture Pro (version 2.8.8, Jenoptik, Jena, Germany) and were recorded throughout five early- and latewood cells along five cell rows (i.e., *n* = 25 cells per tree), and the mean values and standard deviations were calculated. The proportion of cell wall material was determined as the ratio between CLD and CWT. Student’s independent sample *t*-test was used to determine significant differences among control and girdled trees with respect to determined cell parameters.

## Results

**Figure 1:**
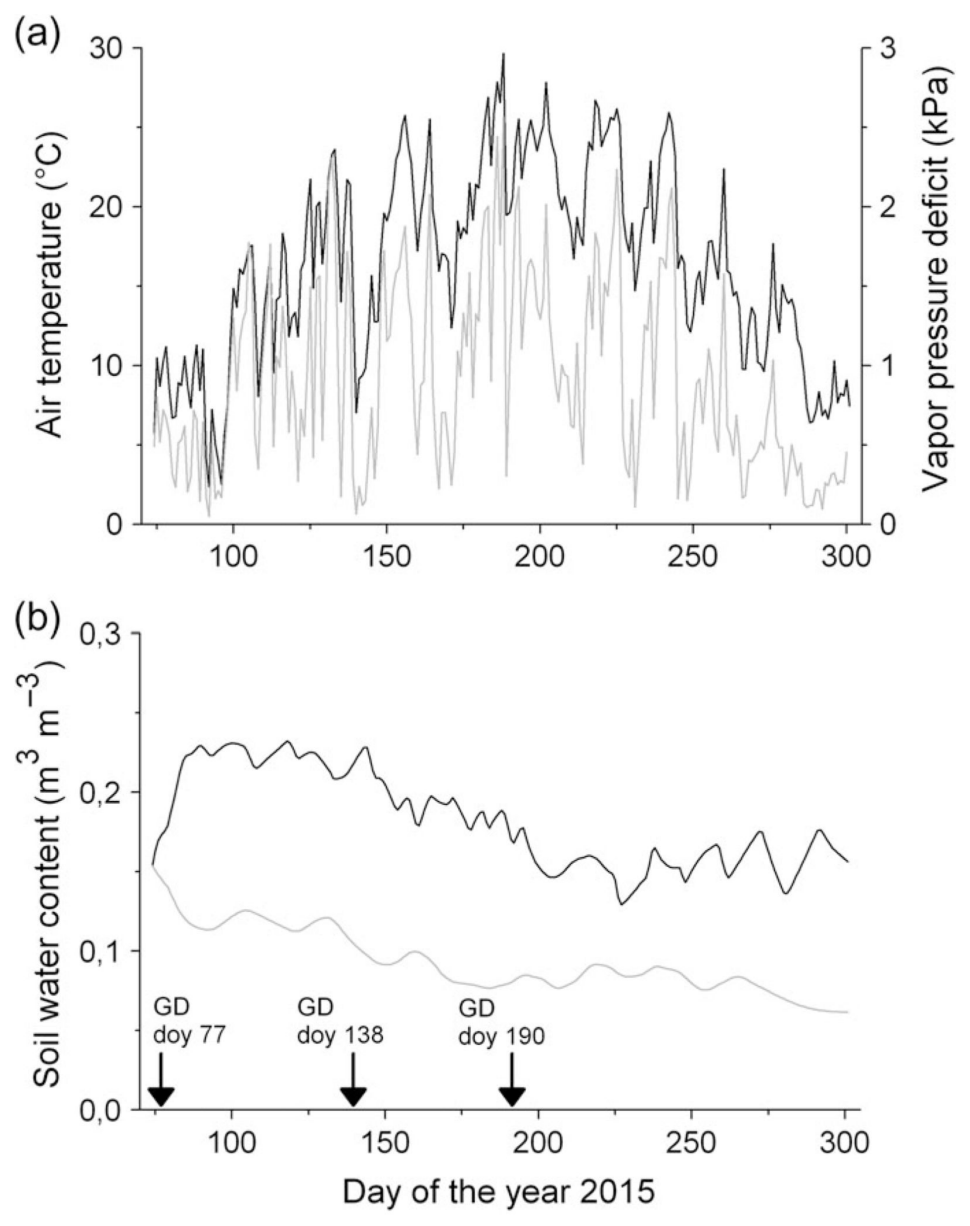
Environmental conditions in the polytunnel during the growing season 2015. (a) Air temperature (black line) and vapor pressure deficit (gray line). (b) Soil water content (10 day moving averages) in watered (black line) and drought-stressed trees (gray line). Arrows indicate girdling dates (GD; doy = day of the year).

In the polytunnel, the mean daily air and soil temperature during April through October amounted to 18.1 ± 5.6 °C and 18.4 ± 5.3 °C (both SH-treatments), respectively (Figure 1a and data not shown). The mean daily vapor pressure deficit during the study period ranged between 0.05 and 2.57 kPa (mean = 0.92 ± 0.6 kPa; Figure 1a) and the mean daily maximum solar radiation (PhAR) was 1005 μmol m^−2^ s^−1^ (data not shown). At the start of the experiment the SWC averaged 0.14 ± 0.02 and 0.15 ± 0.02 m^3^ m^−3^ in the watered and drought-stressed treatments, respectively. The mean growing season SWC values were 0.09 ± 0.02 m^3^ m^−3^ in the drought-stressed and 0.18 ± 0.04 m^3^ m^−3^ in the watered containers (Figure 1b).

**Figure 2:**
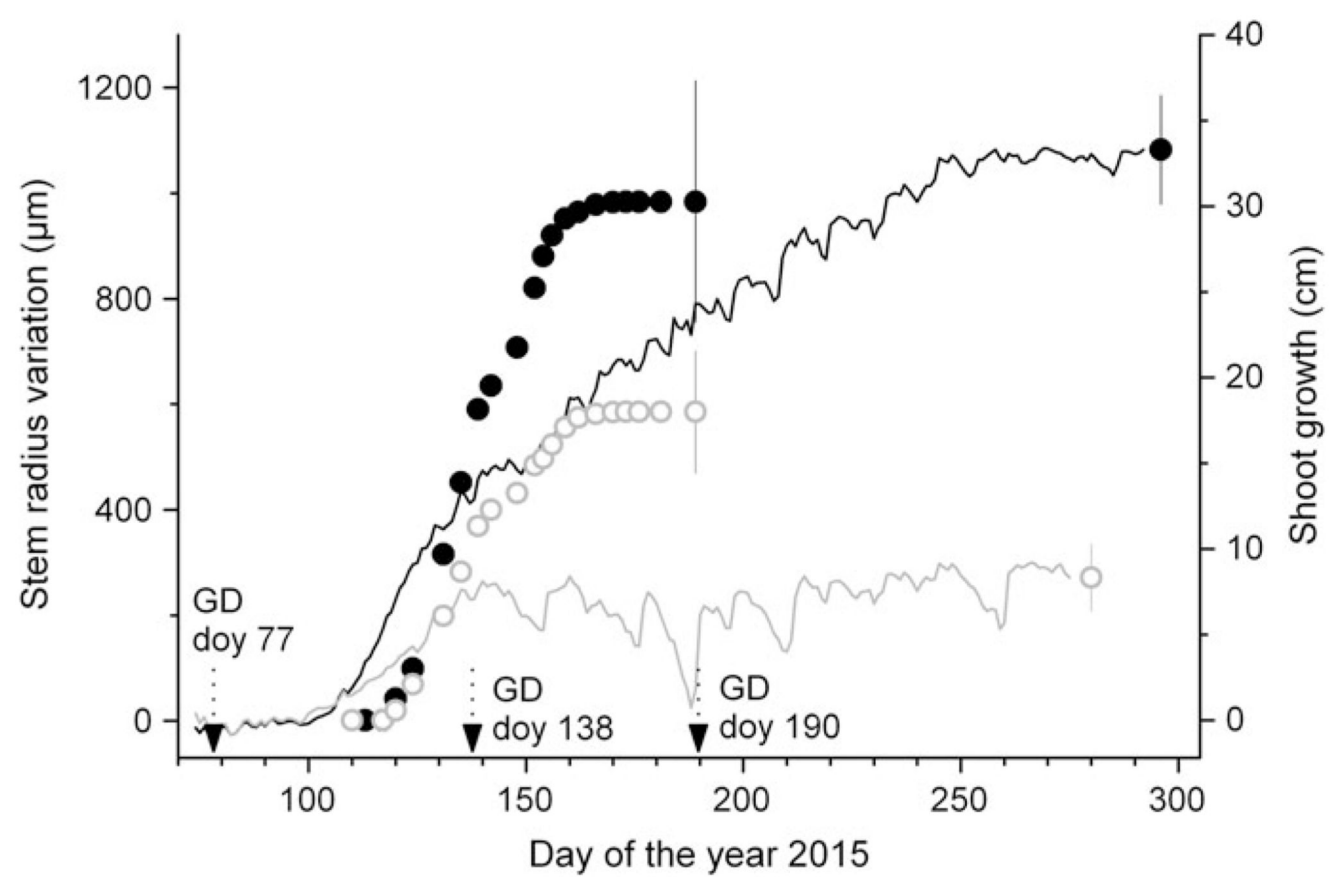
Intra-annual dynamics of radial (lines) and shoot growth (symbols) in watered (black line and filled circles) and drought-stressed (gray line and open symbols) non-girdled trees. Bars indicate mean standard deviation among records. Arrows indicate girdling dates (GD; doy = day of the year).

In both SH-treatments radial growth started in early April (about doy 100) and ceased approximately at the end of May (doy 150) and mid-September (doy 260) in drought-stressed and watered trees, respectively. Growth of the terminal leader started in May and had already ceased in early June in watered and drought stressed trees (doy 160; Figure 2). Hence, while radial growth lasted for ~6–7 weeks in drought-stressed trees and ~22 weeks in watered trees, shoot growth duration amounted to ~6 weeks in both SH-treatments. Statistically significant reductions (*P* ≤ 0.001) of radial growth by ~65% and leader shoot growth by ~40% in the drought-stressed compared with watered trees indicate a pronounced SH-treatment effect.

**Figure 3:**
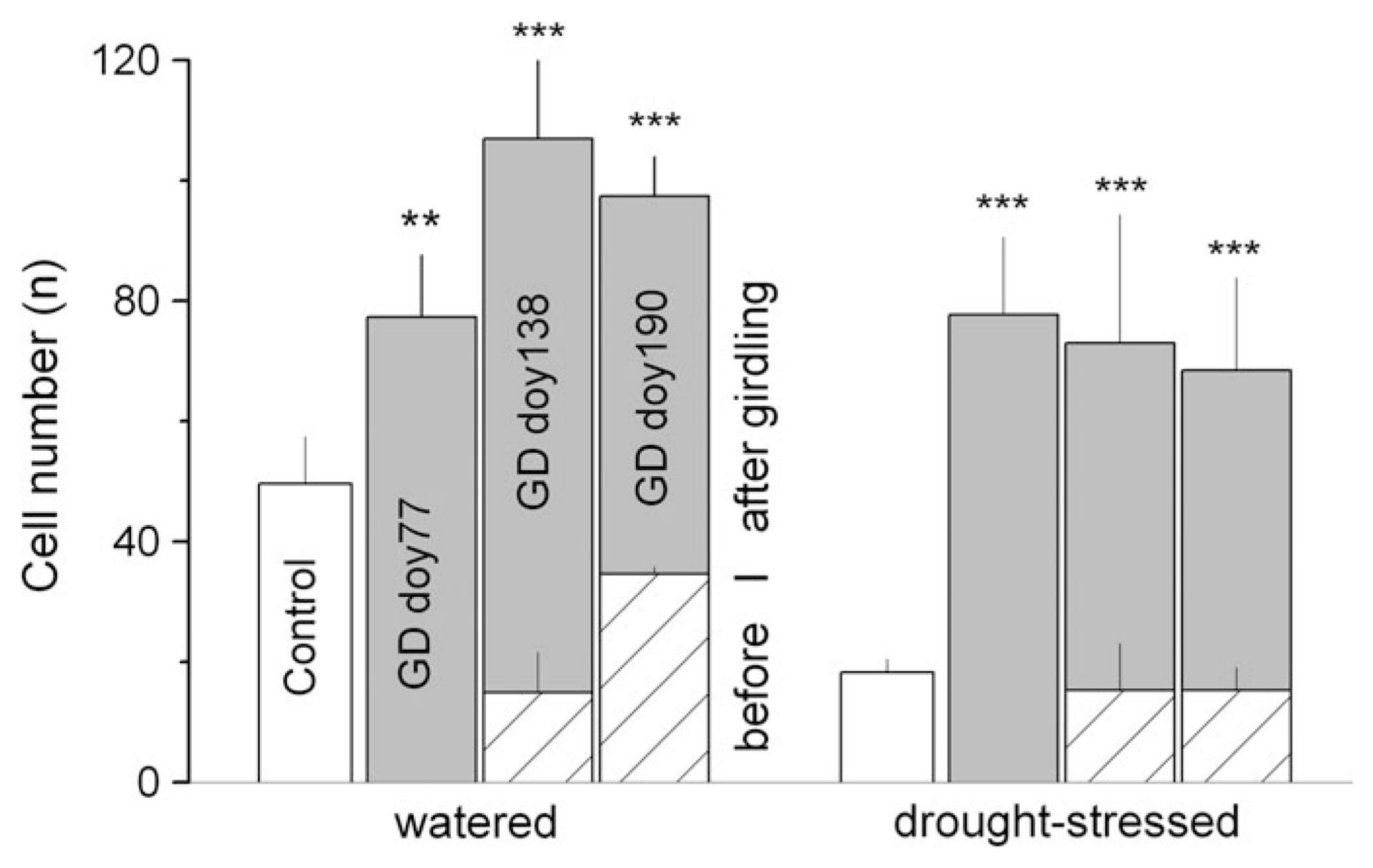
Number of tracheids in controls and above the girdling zone in watered and drought-stressed trees. Cell numbers developed before and after girdling are indicated by stacked hatched and gray bars, respectively. Standard deviations are indicated. Asterisks indicate statistical significant difference in total cell number between girdled trees and controls. **P < 0.01; ***P < 0.001. GD = girdling date; doy = day of the year.

In watered controls more than twice as many xylem cells were developed in comparison with non-girdled drought-stressed trees (Figure 3). In response to girdling the total cell number in UZ significantly increased irrespective of SH-treatment and GD. Non-significant differences in the number of tracheid cells developed before girdling (GDs doy 138 and doy 190) in drought-stressed compared with control trees indicate that radial growth in the former group ceased at the end of May, which is consistent with dendrometer records (cf. Figure 2). In contrast to striking cambial response in UZ a lack of tracheid cell formation was observed below girdling, i.e., in MZ and LZ after all GDs and in both SH-treatments.

**Figure 4:**
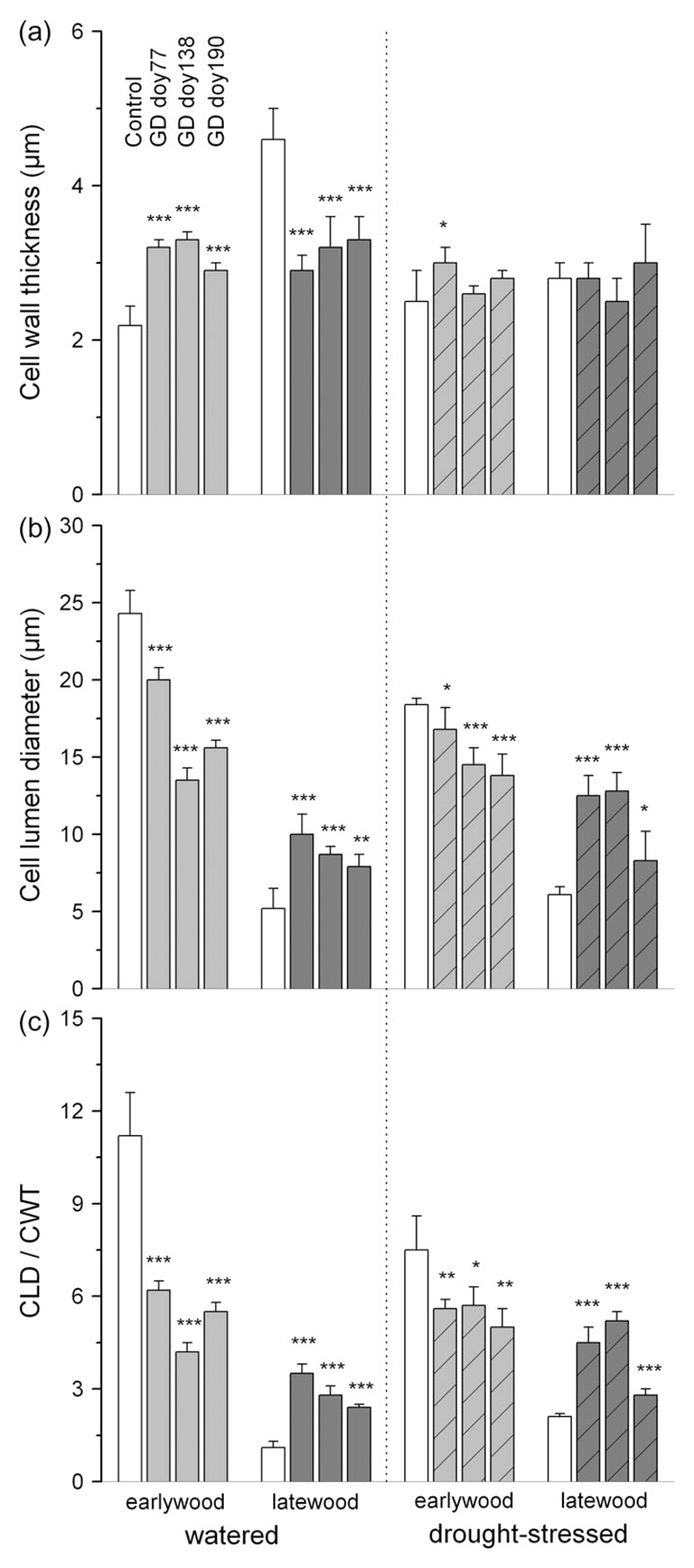
Wood anatomical parameters of earlywood and latewood: cell wall thickness (CWT) (a); cell lumen diameter (CLD) (b); ratio of CLD and CWT (c) in controls (open bars) and girdled watered and drought-stressed trees (hatched bars). Light gray and dark gray bars denote earlywood and latewood, respectively. Standard deviations are indicated. Asterisks indicate statistical significant difference in wood anatomical parameters between girdled trees and controls. *P < 0.05; **P < 0.01; ***P < 0.001. GD = girdling date; doy = day of the year.

In non-girdled controls lack of water supply caused significant reduction of CLD in earlywood, and CWT in latewood (*P* < 0.001; Figure 4). Correspondingly, the ratio of CLD:CWT was significantly lower in earlywood (*P* = 0.002) and significantly higher in latewood (*P* < 0.001) of trees exposed to drought stress compared with watered trees. In control trees, CWT and CLD in earlywood and latewood, respectively, were not significantly different among SH-treatments (*P* > 0.1).

Irrespective of SH-treatment and GD, wood produced after girdling significantly differed from ‘normal’ wood, i.e., wood in non-girdled controls (Figure 4). Analyses of wood anatomical parameters revealed that above girdling, CWT of earlywood and latewood significantly increased and decreased, respectively, in watered trees at all GDs (Figure 4a). In drought-stressed trees CWT significantly increased in earlywood in response to girdling at GD doy 77 only, while CWT of latewood showed no significant change in girdled trees subjected to drought. In both SH-treatments CLD significantly decreased and increased in earlywood and latewood, respectively, compared with controls (Figure 4b). The proportion of cell wall material (CLD:CWT ratio) incorporated into tracheids significantly increased in earlywood and decreased in latewood of girdled trees. The significant decrease in CLD:CWT ratio in the earlywood was due to an increase in CWT and a decrease in CLD, while the significant increase in CLD:CWT ratio in the latewood was primarily caused by a striking increase in CLD, which almost doubled in the watered and more than doubled in the drought-stressed trees compared with the control trees (Figure 4). In trees girdled at GD doy 77 a significant increase (*P* < 0.001) in CLD and CLD:CWT ratio in the latewood of the watered and drought-stressed trees was also detected 50 cm above UZ (data not shown). In Figure S2a and b, available as Supplementary Data at *Tree Physiology* Online, latewood cross-sections of a control and girdled watered tree are shown. In the girdled tree significantly wider latewood conduits with thinner cell walls (cf. Figure 4a and b) were developed compared with the control, which was not in agreement with the Mork-latewood criterion, i.e., double CWT ≥ CLD (Mork 1928).

**Figure 5:**
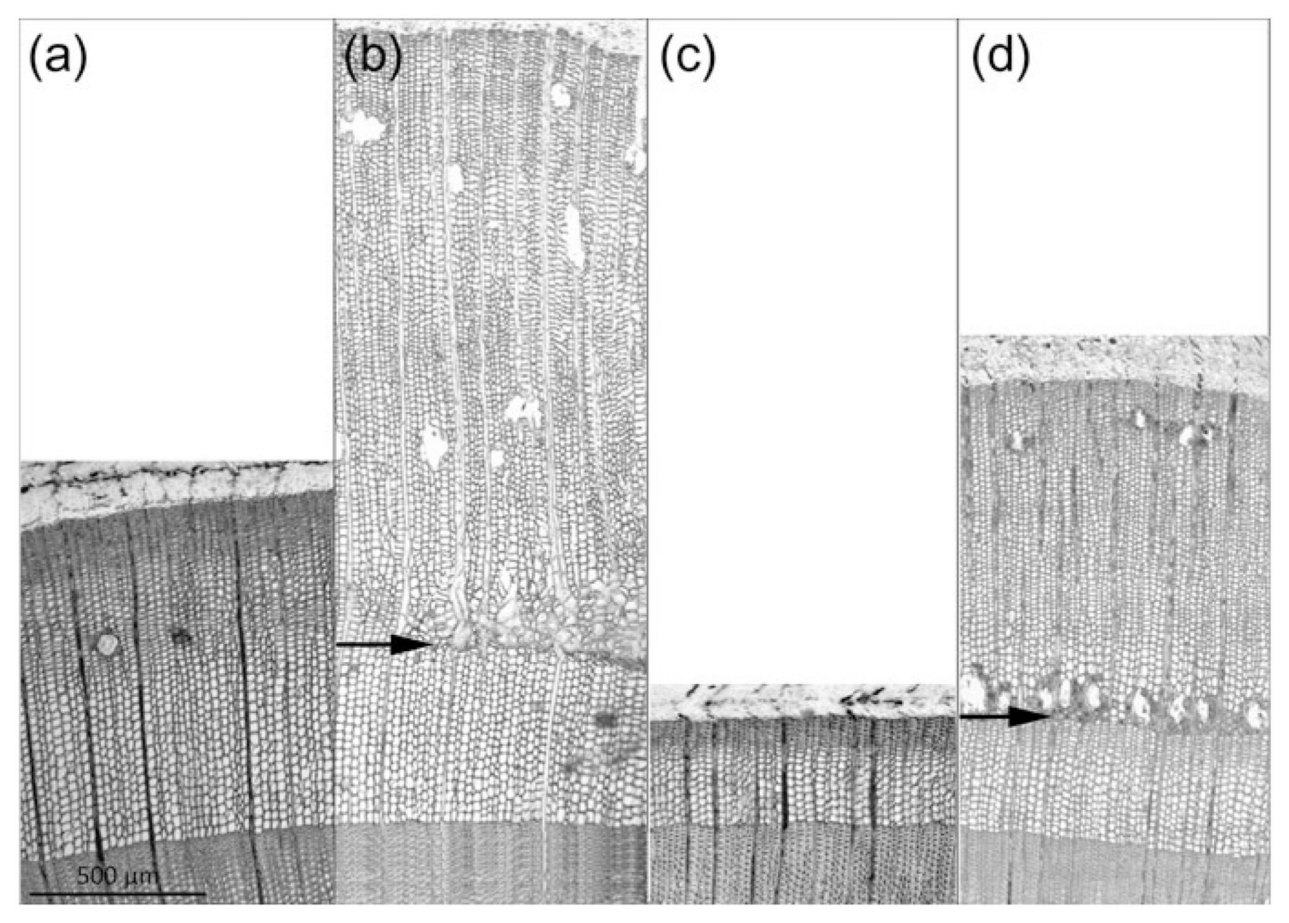
Transverse stems sections of watered control (a) and girdled tree (b) and drought-stressed control (c) and girdled tree (d). Girdling occurred during earlywood formation on doy 138 in the watered tree and on doy 190, i.e., about 6 weeks after growth cessation (i.e., latewood completely developed) in the drought-stressed tree. Proliferating ray parenchyma cells and tangential resin canals mark time of girdling in the watered (b) and drought-stressed tree (d), respectively (see arrows).

Influence of girdling on wood formation in watered trees during vigorous earlywood growth (GD doy 138) and in drought-stressed trees >6 weeks after end of the growing season (GD doy 190) is exemplary depicted in Figure 5a–d, respectively. In the watered tree girdling during earlywood formation caused striking increase of cambial cell division in the zone above girdling compared with control (Figure 5a and b). Several layers of thick-walled latewood cells indicate growth cessation at the time of girdling in July (GD doy 190) in drought-stressed trees (Figure 5c; cf. Figure 2). Girdling caused reactivation of cambial activity and intensive cell production above the girdling zone (Figure 5d).

## Discussion

### Effects of drought on tree growth and wood anatomy

The direct effects of water availability on tree growth and wood anatomy are well-known phenomena (e.g., Hsiao 1973, Schweingruber 2007, Fonti et al. 2010, Balducci et al. 2015). Consistent with these findings, radial and leader shoot growth in drought-stressed non-girdled *P. abies* saplings were significantly reduced compared with watered trees. Drought stress also strikingly shortened the period of radial growth, which is in agreement with reports of several authors that cambial activity stops under severe drought (Arend and Fromm 2007, Gruber et al. 2010, Eilmann et al. 2011, Ren et al. 2015, Balducci et al. 2016). The most conspicuous anatomical changes of drought were a significant decrease of CLD (expansive growth) in earlywood and CWT (increase in biomass) in latewood. The decrease in CLD under drought is related to diminished cell enlargement caused by a lack of water supply (Hsiao 1973, Nicholls and Waring 1977, Sheriff and Whitehead 1984, Sterck et al. 2008) and/or C availability for maintaining turgor pressure via osmotic potentials (Ray et al. 1972, Larcher 2003, Deslauriers et al. 2016). Reduced CWT is most likely caused by diminished C assimilation due to closure of the stomata under drought (Liang and Eckstein 2006, Steppe et al. 2006, Eilmann et al. 2011, Martin-Benito et al. 2013, Deslauriers et al. 2014), which is supported by findings of several authors (Zweifel et al. 2009, Leo et al. 2014, Oberhuber et al. 2015b), who reported stomatal control of tree water status in *P. abies* exposed to soil dryness (isohydric behavior).

### Effects of modified C availability on cambial activity and wood anatomy

The main focus of this study was to investigate effects of interrupted C flow at different phenological stages on cambial activity and wood anatomical parameters in watered and drought-stressed *P. abies* saplings. Due to blocking of the downward translocation of soluble sugars, accumulation and depletion of carbohydrates above and below the girdle, respectively, was reported to occur (Högberg et al. 2001, Daudet et al. 2005, Peuke et al. 2005, Maier et al. 2010). The accumulation of carbohydrates above girdling was found to stimulate radial growth (Wilson 1968, Noel 1970, Daudet et al. 2005, De Schepper et al. 2010, De Schepper and Steppe 2011), while below girdling radial growth stops (Maier et al. 2010, Maunoury-Danger et al. 2010, De Schepper and Steppe 2013). In agreement with these studies and our expectation, we found a significant increase in cambial cell division above the girdling zone and a lack of cell formation below. Hence, the subsequent discussion on effects of phloem girdling on wood formation is related to the zone above girdling, i.e., UZ.

Irrespective of SH-treatment, blockage of C transport in the phloem caused a significant increase in CWT of the earlywood cells, which is in accordance with the observations of Deslauriers et al. (2016), who considered C availability as a constraint that is directly involved in building the cell wall of *Picea mariana* saplings. It is well known that cambial activity and xylem cell development are considerable energy sinks and depend on a continuous supply of carbohydrates (Hansen and Beck 1994, Oribe et al. 2003, Muller et al. 2011). Therefore, we explain the decrease in CWT in latewood in watered trees by the striking increase in number of tracheid cells, an increase in maintenance respiration (cf. Domec and Pruyn 2008) and most likely production of phenolic and tannin compounds as a wound response to girdling (cf. Maunoury-Danger et al. 2010). Hampered C supply due to feedback inhibition, i.e., downregulation of photosynthesis (e.g., Iglesias et al. 2002, Vemmos et al. 2012, López et al. 2015) or the transfer of excess sugars to storage components (Maier et al. 2010, Pantin et al. 2013), can be excluded as we observed no significant decrease in the content of non-structural carbohydrates (starch and soluble sugars) in the shoot in response to girdling (cf. Oberhuber et al. 2017).

While a tremendous increase in cambial cell division occurred irrespective of water availability, tracheids with significantly narrower lumen diameters were formed in the earlywood in response to girdling, which is in contrast to our hypothesis. We suggest that a shortage or absence of some internal factor(s) or hormonal imbalance restricted the enlargement of cambial derivatives during differentiation of earlywood tracheids. Although the decline of CLD caused a reduction of hydraulic conductivity (Tyree and Zimmermann 2002), it is outweighed by the striking increase in tracheid cell number in both SH-treatments, which leads to a larger conducting area. On the other hand, striking increase in CLD in latewood in both SH-treatments might be due to successive accumulation of osmotically active sugars during the growing season, which increase osmotic pressure according to the van’t Hoff equation (Jones 1992) and cause more water to be drawn into the cambial zone. Furthermore, our results are in accordance with a modeling study by De Schepper and Steppe (2011), who found that changes in turgor pressure due to changes in sugar concentrations were the key driving variable of cell division and expansion in response to girdling. As a consequence of significant increase in CLD (both SH-treatments) and decrease in CWT (watered trees) in latewood, no latewood according to Mork (1928), i.e., CLD ≤ double CWT, was developed after girdling in watered and drought-stressed trees. An increase in the ratio CLD:CWT indicates a decrease in wood density, which is related to higher hydraulic conductivity at the expense of lower cavitation resistance (e.g., Pittermann et al. 2006, for reviews see Rosner 2013 and Hacke et al. 2015). Girdling blocks C transport to belowground and accumulation of osmotically active sugars might induce a delay in transition from earlywood to latewood. Our reasoning is in agreement with findings of Oribe et al. (2003) and Rahman et al. (2016), who reported that cambial cell differentiation is inhibited when there is a depletion of stored carbohydrates near the cambium. Although the involvement of auxin (indole-3-acetic acid, IAA) in the control of latewood formation has frequently been put forward (e.g., Little and Savidge 1987, Funada et al. 2001, Baba et al. 2011), Uggla et al. (2001) reported that initiation of latewood formation in *Pinus sylvestris* is not a consequence of decreased IAA concentrations in dividing and expanding cells. Rather, the authors suggested a role for sugar signaling during earlywood/latewood transition, which might also apply in this study.

### Cambial reactivation in drought-stressed trees in response to girdling

In contrast to our expectations, intense cambial cell division in drought-stressed trees occurred after GDs doy 138 and doy 190, when cambial activity and shoot growth has already ceased. Several authors reported that localized stem heating can initiate cambial cell division in *P. abies* and other conifers during dormancy (e.g., Oribe and Kubo 1997, Oribe et al. 2001, Gričar et al. 2006) due to occurrence of IAA in cambial tissues (Eklund et al. 1998). However, osmotically active C compounds are required to generate water turgor pressure during cell expansion and to release the hydromechanical limitation of growth (Deslauriers et al. 2009, Pantin et al. 2013, Steppe et al. 2015). Because sugar signaling is known to promote cell division (Smith and Stitt 2007, Lastdrager et al. 2014) we therefore suggest that a specific C-based ‘signaling compound’ (sugar- and/or auxin-based) together with an increase in C availability due to blockage of C transport in the phloem are the triggers not only for tremendous increase in cambial cell division but also reactivation of cambial cell activity determined in *P. abies* saplings.

## Conclusions

Both watered and drought-stressed trees showed striking increase in cambial activity in response to girdling and predominantly similar changes in wood anatomical parameters, suggesting C limitation of wood formation in *P. abies* saplings due to high C demand belowground. Although this study highlights the importance of C availability for wood formation and mitigation of drought effects on cambial cell division and differentiation, it is plausible that girdling induced physiological changes unrelated to tree C status (Asao and Ryan 2015). Hence, investigations including plant hormone signaling and phloem transport processes are necessary to conclusively elucidate the specific circumstances yielding the results found in this study.

## Supplementary Data

Supplementary Data for this article are available at *Tree Physiology* Online.

Fig. S1

Fig. S2
