# Supplementary figures and images for: Cambial response of Norway spruce to modified carbon availability by phloem girdling
Source: Tree Physiol. Author manuscript; Available in PMC 2017 Dec 6. (PMC5718295; doi:10.1093/treephys/tpx077)

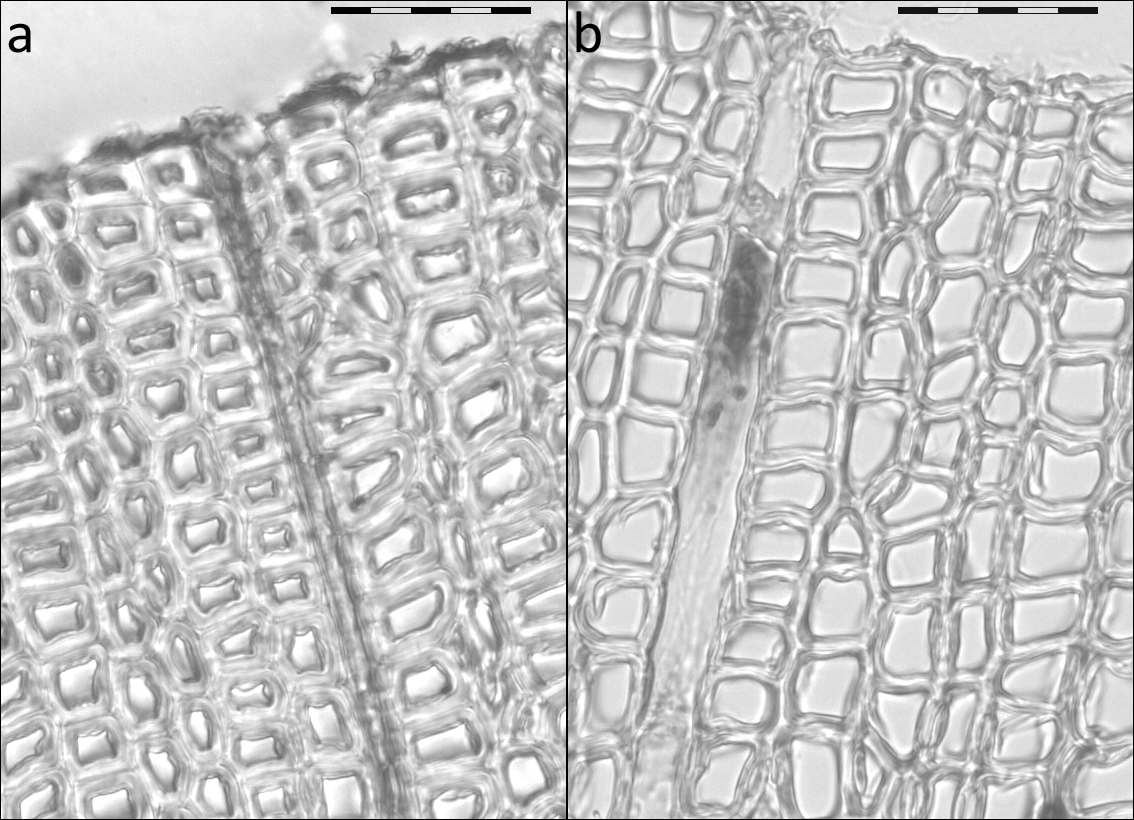

Supplement: Fig. S2 [file NIHMS75195-supplement-Fig__S2.tif]
